# Endogenous polysaccharide–protein composite nanoparticles prepared from spent coffee grounds as multi-flavor carriers

**DOI:** 10.1016/j.fochx.2026.104636

**Published:** 2026-10-15

**Authors:** Haitao Tao, Weilong Guan, Haibo Zhu, Qiuyu Han, Mingyang Dai, Xin Gao, Yawen Lin, Yue Zhang

**Affiliations:** a China Tobacco Jiangsu Industrial Co., Ltd., Nanjing 210019, China; b School of Food Science and Biotechnology, Zhejiang Gongshang University, Hangzhou 310018, China; c Nantong Cigarette Filter Co., Ltd., China Tobacco Jiangsu Industrial Co., Ltd., Nantong 226014, Jiangsu, China

**Keywords:** Spent coffee grounds, Composite nanoparticles, Flavor encapsulation, Cocoa flavor extract

## Abstract

Spent coffee grounds (SCG), an abundant byproduct of coffee production, are rich in endogenous polysaccharides and proteins, offering considerable potential for the development of waste-derived functional materials. In this study, endogenous polysaccharide–protein composite nanoparticles (CPP NPs) were recovered from SCG through alkaline extraction and investigated as flavor carriers for cigarette-related thermal applications. The effects of extraction pH on the composition, physicochemical properties, structural characteristics, and flavor encapsulation performance of the resulting nanoparticles were systematically evaluated. CPP NPs prepared at pH 11 exhibited the most favorable physicochemical properties and were therefore selected for the preparation of a freeze-dried multi-flavor powder (CPP-D5) with both original coffee-derived and extraneous cocoa-related flavors. Major cocoa-related volatile compounds remained detectable in the resulting powder after storage, while their normalized relative proportions varied with storage temperature. Py-GC–MS analysis showed that cocoa-related volatile compounds persisted under moderate thermal conditions, while additional volatile compounds were generated at higher temperatures, increasing the complexity of the volatile profile. These findings suggest that CPP NPs function not only as efficient flavor delivery vehicles but also as active contributors to aroma development during heating. This work demonstrates a high-value utilization strategy for spent coffee grounds and highlights their potential as multifunctional flavor delivery systems for food applications.

## Highlights

• Endogenous polysaccharide–protein nanoparticles were recovered from spent coffee grounds.
• Nanoparticles prepared at pH 11 showed favorable physical properties and flavor encapsulation performance.
• The nanoparticles protected volatile compounds and prolonged aroma retention.
• Heating triggered flavor release and enhanced aroma complexity in food systems.

## 1 Introduction

Spent coffee grounds (SCG), the primary byproduct generated during coffee brewing and instant coffee production, have attracted significant attention owing to the massive global consumption of coffee. It is estimated that approximately 2 kg of wet SCG are generated for every kilogram of instant coffee produced (Piasek et al., 2026). Given the substantial quantities of SCG produced annually, increasing efforts from the industry and academy have been devoted to their valorization. While early research primarily focused on applications such as animal feed, composting, and biofuel production, recent studies have increasingly explored high-value utilizations, including the recovery of polyphenols, dietary fiber, lipids, and other bioactive compounds for food, nutraceutical, and biomaterial applications (Ahmed et al., 2024; Campos-Vega et al., 2015).

SCG contains abundant carbohydrates, proteins, and phenolic compounds, with carbohydrates representing the major fraction, representing 57–82% of the dry matter depending on the coffee source and processing conditions (Salazar-López et al., 2020). These carbohydrates are mainly present as polysaccharides such as cellulose, hemicellulose, pectin, and galactomannans (Lin et al., 2025), whose efficient and high-value utilization holds great promise. In our previous study, polysaccharide–rich fractions were isolated from SCG using different extraction methods, including a commercially applicable process, and their physicochemical properties were compared. Compared with the hot-water and acid extraction methods evaluated previously, alkaline extraction produced a polysaccharide–protein complex fraction with the highest carbohydrate content (52.33 ± 0.59%) and a relatively high protein content (13.06 ± 0.07%), although acid extraction resulted in the highest overall extraction yield (Han et al., 2025). This polysaccharide–protein complex fraction also exhibited superior emulsifying properties, thermal stability, and DPPH radical-scavenging activity, potentially because of its relatively high phenolic content (7.40 ± 0.26%). These phenolic compounds are likely responsible not only for the enhanced antioxidant activity observed in the fraction but also for the development of desirable aroma characteristics during thermal processing, as some phenolics may serve as precursors of aroma-active volatile phenols upon heating (Zakidou et al., 2021). These unique compositional features suggest that this polysaccharide–protein complex may function as both structural matrix and active aroma component for flavor encapsulation and delivery in heat-treated products. Using protein–polysaccharide composite nanoparticles to protect and deliver flavors and other bioactive compounds has become a common strategy (Liu, Chen, et al., 2026). But here, the use of endogenous polysaccharide–protein composite nanoparticles (CPP NPs) derived from SCG is not intended solely to protect and stabilize the incorporated flavor/aroma compounds. The SCG-derived matrix itself can also contribute intrinsic coffee-derived aroma compounds, thereby providing an additional flavor function while simultaneously valorizing SCG as an underutilized food by-product.

Cigarette and other tobacco-related products represent an important application scenario for flavor delivery systems because flavoring compounds (such as menthol, vanillin) are commonly incorporated to modulate and improve sensory characteristics, while many of these compounds are relatively volatile and may undergo substantial loss or transformation during thermal processing (Liu, Huang, et al., 2026). The pyrolysis characteristics of these flavor ingredients, with and without carriers, under various heating and burning conditions are also important for the development of tobacco and other thermally related products (Wang et al., 2025). Recently, increasing efforts have been devoted to exploring the use of biomass-derived materials to develop sustainable flavor-delivery systems for tobacco-related products, with the aim of reducing environmental impacts while maintaining the desired flavor and aroma characteristics. Therefore, developing effective carriers for the controlled delivery and stabilization of flavor compounds under thermally relevant conditions using CPP NPs derived from SCG is of practical interest to the tobacco industry.

Nevertheless, previous research mainly compared bulk polysaccharide-rich fractions obtained using different extraction methods and evaluated their potential in food-packaging materials. The effects of alkaline extraction pH on the formation, composition, and interfacial properties of SCG-derived CPP NPs, particularly their performance as flavor carriers, remain insufficiently understood. Therefore, in this study, SCG-derived CPP NPs were investigated as multifunctional carriers for flavor compounds, with the potential to provide both flavor protection and additional intrinsic coffee-related flavor. Particular emphasis was placed on evaluating the influence of alkaline extraction pH on the physicochemical properties, structural characteristics, and flavor encapsulation performance of the nanoparticles. The nanoparticle formulation with favorable physicochemical properties was subsequently selected for the preparation of freeze-dried flavor powders, and its aroma retention and release behaviors during storage and thermal processing were further examined.

## 2 Materials and methods

### 2.1 Materials

Wet spent coffee (*Coffea arabica* L.) grounds (SCG), generated after brewing with an espresso coffee machine (Suzhou Dr. Coffee System Technology Co., Ltd., China), were obtained from a local coffee shop in Hangzhou, China. *Coffea arabica* L. was selected because it is widely used in specialty coffee shops and is generally associated with a more desirable and complex flavor profile than *Coffea canephora*. Immediately after collection, the wet SCG were packed in sealed polyethylene bags, transported to the laboratory within approximately 1 h at ambient temperature, and immediately subjected to drying upon arrival. The SCG were dried at 105 °C to constant weight. The final moisture content of the dried SCG was approximately 5.0% (*w*/w). The dried SCG were then defatted using n-hexane and stored in a desiccator (RH 0%) until further use (Han et al., 2025). The flavors were the products from Jiangsu Xinyuan Tobacco Sheet Co., Ltd. to be used in the cigarette formula, while the thyme oil and fish oil are obtained from Thermo Fisher Scientific Inc. (Cleveland, OH, USA). Other chemicals and reagents used in this study were purchased from Macklin Inc. (Shanghai, China).

### 2.2 Preparation of endogenous polysaccharide–protein composite nanoparticles (CPP NPs) from SCG

The extraction of polysaccharide–protein composites from SCG was conducted based on the previous study using alkaline extraction method under various pH conditions (Han et al., 2025). The dried SCG powder was mixed with NaOH solution at a ratio of 1:20 (w/v) at different pH values (8, 9, 10, 11), and heated to 95 °C for 3 h. After cooling to 27 ± 1 °C, the suspension was centrifuged using a refrigerated centrifuge (CF16RXII, Hitachi, Japan) at 4000 ×*g* for 15 min at 4 °C. The supernatant was collected and concentrated to 25% of its original volume using a rotary evaporator (R250, Senco Technology Co., Ltd., Shanghai, China). After washing and reprecipitation with an equal volume of ethanol three times, the ethanol precipitate was collected using the same centrifuge at 4000 ×*g* for 15 min at 4 °C. The precipitates prepared by different pH conditions were then redispersed in deionized water and dialyzed using a 3500 Da molecular weight cut-off dialysis bag for 3 days, with the water in outer beaker replaced every 4 h. Subsequently, the dialyzed samples were pre-frozen in an ultra-low-temperature freezer (Thermo Fisher Scientific, USA) at −80 °C for 4 h and then lyophilized for 24 h using a freeze dryer (Beijing Sihuan Furui Technology Development Co., Ltd., Beijing, China). The resulting dried polysaccharide-protein composite powders were ground, sealed, and stored in a desiccator (RH 0%) until further use. For use and measurement, the samples were redispersed in aqueous phase at appropriate concentrations.

### 2.3 Chemical and structural characterizations of CPP NPs

#### 2.3.1 Chemical composition

The total sugar content of prepared CPP NPs was determined using the phenol–sulfuric acid method with glucose as the standard (Zhang et al., 2023). The total phenolic content was measured using the Folin–Ciocalteu method, with gallic acid used as the calibration standard (Samsonowicz et al., 2019). Protein content was quantified according to the Kjeldahl method using a nitrogen-to-protein conversion factor of 6.25 (Xiao et al., 2025). The uronic acid content was determined using the *m*-hydroxybiphenyl method, with galacturonic acid employed as the standard solution (Taylor & Buchanan-Smith, 1992).

#### 2.3.2 The molecular weight distribution and monosaccharide fractions

The molecular weight distribution of the samples was analyzed by high-performance gel permeation chromatography (HPGPC) according to the method reported by Yang et al. with slight modifications (Yang et al., 2025). The samples were dispersed in ultrapure water at a concentration of 5.0 mg/mL and filtered through a 0.22 μm microporous membrane prior to analysis. Chromatographic analysis was then performed using an Agilent 1100 Series chromatograph (Agilent Technologies, USA) equipped with a Waters Ultrahydrogel Linear column (7.8 × 300 mm). The column temperature was maintained at 30 °C. A 50 mM sodium sulfate solution was used as the mobile phase at a flow rate of 0.5 mL/min. The molecular weight distribution was determined using a series of dextran standard solutions.

To determine the monosaccharide composition, the sample was accurately weighed and hydrolyzed with 1 mL of 2 M trifluoroacetic acid at 121 °C for 2 h. After hydrolysis, the sample was washed with methanol 2–3 times to remove residual impurities, then dissolved in sterile water and transferred to a chromatographic vial for analysis (Wang et al., 2018). Monosaccharide analysis was performed using a Thermo ICS 5000+ ion chromatography system (Thermo Fisher Scientific, USA) equipped with an electrochemical detector and a Dionex™ CarboPac™ PA20 column (150 × 3.0 mm, 10 μm). The column temperature was maintained at 30 °C. Ultrapure water, 0.1 M NaOH, and 0.1 M NaOH containing 0.2 M sodium acetate (NaAc) were used as mobile phases. The flow rate was set at 0.5 mL/min, and the injection volume was 5 μL.

#### 2.3.3 Structural characterization

The crystalline structures of CPP samples were analyzed via X-ray diffraction (XRD) using a Miniflex 600 diffractometer (Rigaku, Japan). The measurements were performed with Cu-Kα radiation in step-scanning mode with voltage set at 40 kV, diffraction angle (2θ) range 5–80°, and scanning speed 5°/min, respectively.

The FTIR measurement was performed to characterize the interactions of protein and polysaccharide molecules in the CPP samples. For the FTIR analysis, 2 mg of each sample was mixed with 150 mg of KBr powder, and ground into a uniform fine powder. The resulting mixture was then pressed into a pellet to form a thin, flat film, and launched into Nicolet iS50 FTIR Spectrometer (Thermo Fisher Scientific Instrument, USA). Spectra were recorded in the range of 4000–400 cm^−1^ with a resolution of 4 cm^−1^ and a total of 32 scans.

The thermal stability and thermal decomposition behavior of CPP samples were evaluated using a TGA2 thermogravimetric analyzer (Mettler Toledo, Switzerland) under an inert atmosphere, with high-purity nitrogen used as the protective gas at a flow rate of 50 mL/min.

### 2.4 Characterization of physicochemical properties of CPP NPs

#### 2.4.1 Particle size and zeta potential

The particle size distribution and zeta potential characteristics of CPP particles in aqueous phase were characterized using dynamic light scattering (DLS) by Anton Paar Litesizer 500 (Graz, Austria). The samples were dispersed in ultrapure water at 0.1 mg/mL and filtered through a 0.22 μm microporous membrane before measurement. The analysis was conducted at 25 °C.

#### 2.4.2 Contact angle

The samples (0.2 g) were accurately weighed and pressed into uniformly thick sheets. The sheets were then placed at the bottom of a transparent glass measuring cell containing soybean oil and monitored using a DSA25 goniometer (Kruss, Germany). Ultrapure water was loaded into a microsyringe, and the needle was lowered close to the bottom of the cell without touching the sample surface. The water was then slowly injected into the oil phase at a rate of 2 μL/s, allowing the water droplets to gradually generate at the oil/sample interface. The entire process was monitored, and the three-phase contact angle was determined by fitting the Young–Laplace equation (Panzella et al., 2017).

#### 2.4.3 Interfacial tension

The interfacial tension measurements were performed using the goniometer described above (Feng, Zhang, et al., 2023). The CPP samples were dispersed in the aqueous phase at 2 mg/mL to 10 mg/mL and mixed overnight to ensure full hydration. After loading the sample into a syringe (needle outer diameter = 1.8 mm), the U-shaped syringe was inserted into a glass cuvette filled with soybean oil or fish oil. A 20 μL droplet was formed at the interface, and the dynamic interfacial tension was monitored for up to 5 min. The air/water surface tension was measured using the pendant drop method directly.

### 2.5 Characterization of emulsifying properties of CPP NPs

The CPP samples were dispersed in water at concentrations of 0.1%, 0.5%, and 1% (w/v) and stirred overnight at 600 rpm at 27 ± 1 °C. The pH was adjusted to 6 before mixing with 10% soybean oil or 1% (v/v) fish oil, followed by homogenization for 5 min at 12,000 rpm using a T18 high-speed homogenizer (IKA, Staufen, Germany) to obtain the emulsions. The droplet size of emulsions was measured using SYNC laser diffraction particle size analyzer (Microtrac, Pennsylvania, USA) at 25 °C. The emulsion samples were diluted 100 times with deionized water before measuring. The refractive index of the dispersion medium (water) was set to 1.33, and the refractive index of the emulsion droplets was set to 1.47. The results are expressed as the volume average diameter (d4,3). The zeta potential of emulsions was measured using Anton Paar Litesizer 500 (Graz, Austria) with the emulsion diluted 100 times. The stability of the emulsions was evaluated by measuring the zeta potential and droplet size distribution on days 0, 3, and 7 after preparation.

In another set of experiments, six commercial flavor compounds or formulated products, including menthol, vanilla bean flavor, vanillin, geraniol, cocoa flavor extract, and vanilla extract, were selected as representative flavoring ingredients based on their common use in cigarette-related products and their relevance to thermally processed applications. In this study, “multi-flavor” refers to the overall applicability/flavor profile, not necessarily co-loading multiple individual flavors in every formulation. The CPP sample, labeled as CPP-11 (prepared at 95 °C for 3 h with a solid-to-liquid ratio of 1:20 w/v, pH 11) was formulated into a 1% (w/v) aqueous solution and stirred overnight at 27 ± 1 °C at 600 rpm to ensure complete mixing. Subsequently, the pH of the solution was adjusted to 6, and each flavor product was added at a volume ratio of 1% (v/v). The mixtures were then homogenized using an IKA high-speed homogenizer at 12,000 rpm for 5 min to obtain stable emulsions of menthol, vanilla bean flavor, vanillin, geraniol, cocoa flavor extract, and vanilla extract, labeled as D1–D6, respectively. The particle size and zeta potential of these emulsions during 7 days of storage were measured using the methods described above.

### 2.6 Characterization of the CPP NPs-stabilized flavor formulation

#### 2.6.1 SEM observation

The CPP NPs and the CPP NP-stabilized cocoa flavor extract emulsion (D5) prepared above were lyophilized under the same conditions described above. The microstructure/morphology of the freeze-dried composite powders was observed using a scanning electron microscope (SEM; Regulus 8230, Hitachi, Japan). Images were acquired at an accelerating voltage of 5 kV with a magnification of 1700 × .

#### 2.6.2 HS-SPME-GC–MS analysis

Volatile compounds in lyophilized CPP NPs and the lyophilized CPP-D5 formulation, stored at 4, 25, and 45 °C for up to 1 week, were qualitatively analyzed by HS-SPME-GC–MS using an Agilent 7890A–5975C GC–MS system (Agilent Technologies, Santa Clara, CA, USA), and their relative abundances were estimated by peak-area normalization. Briefly, an accurately weighed sample (0.5 g) was placed into a 15 mL headspace vial and sealed. A 50/30 μm DVB/CAR/PDMS fiber was used for extraction after preconditioning at 250 °C. Headspace adsorption was performed at a sample temperature of 80 °C for 40 min, followed by thermal desorption in the GC injection port at 250 °C for 3 min prior to chromatographic separation. Gas chromatographic analysis was conducted using a DB-WAX capillary column (30 m × 0.25 mm × 0.25 μm) with high-purity helium as the carrier gas at a flow rate of 1.0 mL/min under splitless injection conditions. The oven temperature program was as follows: initial temperature of 40 °C held for 2 min, increased to 100 °C at a rate of 5 °C/min, and then increased to 230 °C at 10 °C/min and held for 10 min. The injection port and transfer line temperatures were set at 250 °C and 240 °C, respectively. MS detection was performed using an electron ionization source at 70 eV, with ion source and quadrupole temperatures maintained at 230 °C and 150 °C, respectively. Mass spectra were acquired over an *m*/*z* range of 20–500. Compound identification was carried out by matching spectra with the NIST 23 Mass Spectral Library (National Institute of Standards and Technology, USA) combined with retention time comparison, while siloxane-derived column-bleed peaks were excluded. The relative contents of volatile compounds were calculated using the peak area normalization method to evaluate compositional differences among samples. The HS-SPME-GC–MS analysis was conducted with reference to previously reported procedures for SCG and cocoa volatiles, with appropriate modifications (Andrade et al., 2022; Chóez-Guaranda et al., 2024).

#### 2.6.3 Py-GC–MS analysis

The volatile pyrolysis products of lyophilized CPP NPs and CPP NP-stabilized cocoa flavor extract (CPP-D5) were analyzed using pyrolysis–gas chromatography–mass spectrometry (Py-GC–MS) with an EGA3030D pyrolyzer (Frontier Laboratories, Koriyama, Japan) coupled to a GCMS-QP2020NX system (Shimadzu Corporation, Kyoto, Japan) and expressed as relative abundance (%). Briefly, approximately 0.3 mg of each sample was accurately weighed and placed into a pyrolysis tube for analysis. The pyrolysis temperatures were set at 200, 275, and 350 °C, with a pyrolysis time of 0.2 min. The resulting pyrolysates were directly transferred into the GC system through an injector maintained at 300 °C for separation and analysis. Gas chromatographic separation was performed using an Rtx-5MS capillary column (30 m × 0.25 mm × 0.25 μm). High-purity helium was used as the carrier gas at a constant flow rate of 1.0 mL/min with a split ratio of 40:1. The oven temperature program was as follows: the initial temperature was held at 40 °C for 3 min, increased to 140 °C at a rate of 10 °C/min, followed by a further increase to 310 °C at 20 °C/min and maintained for 8 min. The injector and transfer line temperatures were maintained at 300 °C and 310 °C, respectively. Mass spectrometric detection was carried out using an electron ionization (EI) source operating at 70 eV, with an ion source temperature of 230 °C and a scanning range of *m/z* 29–600. The relative abundance of each detected compound was calculated using the peak-area normalization method as $A_{i}/\sum A_{i}\times 100$, where $A_{i}$ represents the chromatographic peak area of compound i, and $\sum A_{i}$ represents the total peak area of all detected compounds in the same chromatogram. Compound identification was performed by comparison with the NIST 23 Mass Spectral Library (National Institute of Standards and Technology, USA) and retention index (RI) matching, with background interference peaks excluded. The relative abundance of each compound was calculated by dividing its peak area by the total peak area of all detected compounds and expressing the result as a percentage. The relative abundance of each compound was calculated by dividing its chromatographic peak area by the total peak area of all detected compounds and expressing the result as a percentage (Peng et al., 2025).

#### 2.6.4 DPPH radical-scavenging activity before and after thermal treatment

DPPH radical-scavenging activity was determined according to the method described by Guan et al. (2026), with modifications. Lyophilized CPP, free cocoa flavor (Free D5), and CPP-D5 samples were dispersed in deionized water to achieve a concentration of 0.01 g/mL and diluted to 0.50 mg/mL before measurement. All samples were incubated at 25 or 80 °C for 0 to 2 h.

An aliquot of each diluted sample (100 μL) was mixed with 1.0 mL of the DPPH ethanol solution (40 μg/mL) and sonicated in the dark for 10 min. The mixture was then centrifuged at 5000 ×*g* for 10 min, and the absorbance of the supernatant was measured at 517 nm using a UV-5100 UV–Vis spectrophotometer (Shanghai Metash Instruments Co., Ltd., Shanghai, China). The DPPH radical-scavenging activity was calculated using the following equation:

(1) $$\text{DPPH radical}-\text{scavenging activity}\;\left(\%\right)=\left[1-\frac{A_{1}-A_{2}}{A_{0}}\right]\times 100$$

Here, A_1_ is the absorbance of the sample mixed with the DPPH solution, A_2_ is the absorbance of the sample mixed with an equal volume of ethanol, and A_0_ is the absorbance of the DPPH control with an equal volume of water.

### 2.7 Statistical analysis

All samples and experiments in this study were performed in triplicate. The statistical analysis was conducted using SPSS 26.0 (IBM Corp., USA) statistical software. Differences between groups were assessed using one-way ANOVA, followed by post hoc comparisons with Tukey's multiple comparisons test (with *p* < 0.05).

## 3 Results and discussion

### 3.1 The composition and structural characteristics of CPP as affected by extracting pH conditions

As reported in previous study, after alkaline extraction, the obtained extract mainly exists as an endogenous composite material consisting of polysaccharides, proteins, and phenolic compounds, and is present in nanoparticle form in the aqueous phase (Han et al., 2025). Table 1 shows the impact of extraction pH conditions on the chemical composition of the obtained CPP NPs. The product yield increased significantly with increasing pH, reaching the highest yield of 7.00 ± 0.64% at pH 11, as alkali could more effectively disrupt the cell wall structure, thereby releasing more polysaccharide molecules (Sun et al., 2018). The total sugar content increased significantly from 45.71 ± 1.80% at pH 8 to 53.26 ± 2.64% at pH 9 (*p* < 0.05), but subsequently decreased to 44.76 ± 1.07% and 45.29 ± 1.11% at pH 10 and 11, respectively. This pattern may reflect the relatively selective extraction of carbohydrate-rich components at pH 9. At higher pH values, the increased co-extraction of proteins, as indicated by the increase in protein content to 16.07 ± 0.53% at pH 10 and 19.56 ± 1.34% at pH 11, likely reduced the relative proportion of total sugar in the recovered CPP fraction (Amine et al., 2014). A relatively high phenolic compound yield was achieved at pH 10, as excessively high pH conditions may degrade phenolic compounds (Blackwell et al., 2012), thereby reducing the extraction efficiency.

**Table 1:** Chemical compositions of CPP NPs obtained from SCG as affected by extraction pH conditions.

	pH 8	pH 9	pH 10	pH 11
Yield (%)	3.88 ± 0.33^c^	4.27 ± 0.18^c^	5.78 ± 0.20^b^	7.00 ± 0.64^a^
Total sugar (%)	45.71 ± 1.80^b^	53.26 ± 2.64^a^	44.76 ± 1.07^b^	45.29 ± 1.11^b^
Protein (%)	9.46 ± 3.08^c^	13.64 ± 0.09^b^	16.07 ± 0.53^b^	19.56 ± 1.34^a^
Total phenols (%)	5.76 ± 0.08^c^	5.88 ± 0.13^c^	6.50 ± 0.14^a^	6.23 ± 0.22^b^
M_w_ (kDa)	8.95	13.14	14.13	15.28
	
	Sugar Composition (mol mass ratio)			
Ara	13.78%	14.21%	15.58%	17.96%
Rha	1.64%	1.55%	1.70%	1.72%
Gal	49.76%	50.94%	55.61%	62.52%
Man	30.78%	29.61%	23.14%	13.41%
Glc-UA	2.57%	2.56%	2.68%	3.28%

Note: Values for yield, total sugar, protein, and total phenols are presented as mean ± SD (*n* = 3). Different superscript letters within the same row indicate significant differences (*p* < 0.05). Abbreviations: CPP NPs, polysaccharide–protein composite nanoparticles; SCG, spent coffee grounds; M_w_, weight-average molecular weight; Ara, arabinose; Rha, rhamnose; Gal, galactose; Man, mannose; Glc-UA, glucuronic acid.

Hemicellulose is the main carbohydrate component of SCG. Its hydrolysis produces monosaccharides such as galactose, mannose, and arabinose, with galactose generally occurring at a relatively high proportion (Liu et al., 2023). Polysaccharides obtained by alkaline extraction are generally considered to originate primarily from cell-wall hemicellulose (Kim et al., 2016). Our results showed that galactose was the predominant monosaccharide in the NPs, with the highest content reaching 62.52% at pH 11, further confirming the findings of previous studies and demonstrating the effectiveness of alkaline extraction in this study. As the alkalinity of the extraction increased, the average molecular weight of the CPP also increased. Studies have shown that under alkaline pH conditions, OH^−^ ions can hydrolyze the acetyl and glycosidic bonds in hemicellulose, thereby releasing more high-molecular-weight polysaccharide components (Liu et al., 2023).

The structural characteristics of CPP as affected by extraction pH conditions were also investigated using several techniques. As shown in Fig. 1A, all samples exhibited a broad absorption band near 3469 cm^−1^ and a shoulder band around 2926 cm^−1^. These bands were mainly assigned to O—H and C—H stretching vibrations, respectively, and are characteristic of carbohydrates (Ballesteros et al., 2018; Li et al., 2022). The bands associated with the asymmetric stretching of carboxylate groups (COO^−^) were more intense in CPP-10 and CPP-11 (Ballesteros et al., 2018), suggesting a higher uronic acid content in these samples. This is consistent with the higher glucuronic acid (Glc-UA) content shown in Table 1. No obvious characteristic diffraction peaks were observed in the diffraction patterns of all CPP samples (Fig. 1B), suggesting that the composite extracts possess an amorphous structure, rather than crystalline structure. The extraction pH also showed no significant effect on the peak positions in the thermal degradation profiles of the CPP samples (Fig. 1D). However, the weight-loss rate in the TG curves decreased as the extraction pH increased (Fig. 1C). This decrease may result from the removal of low-molecular-weight compounds and the formation of carboxylate salts, thereby enhancing the thermal stability of CPP NPs(Jiang et al., 2020).

**Fig. 1:**
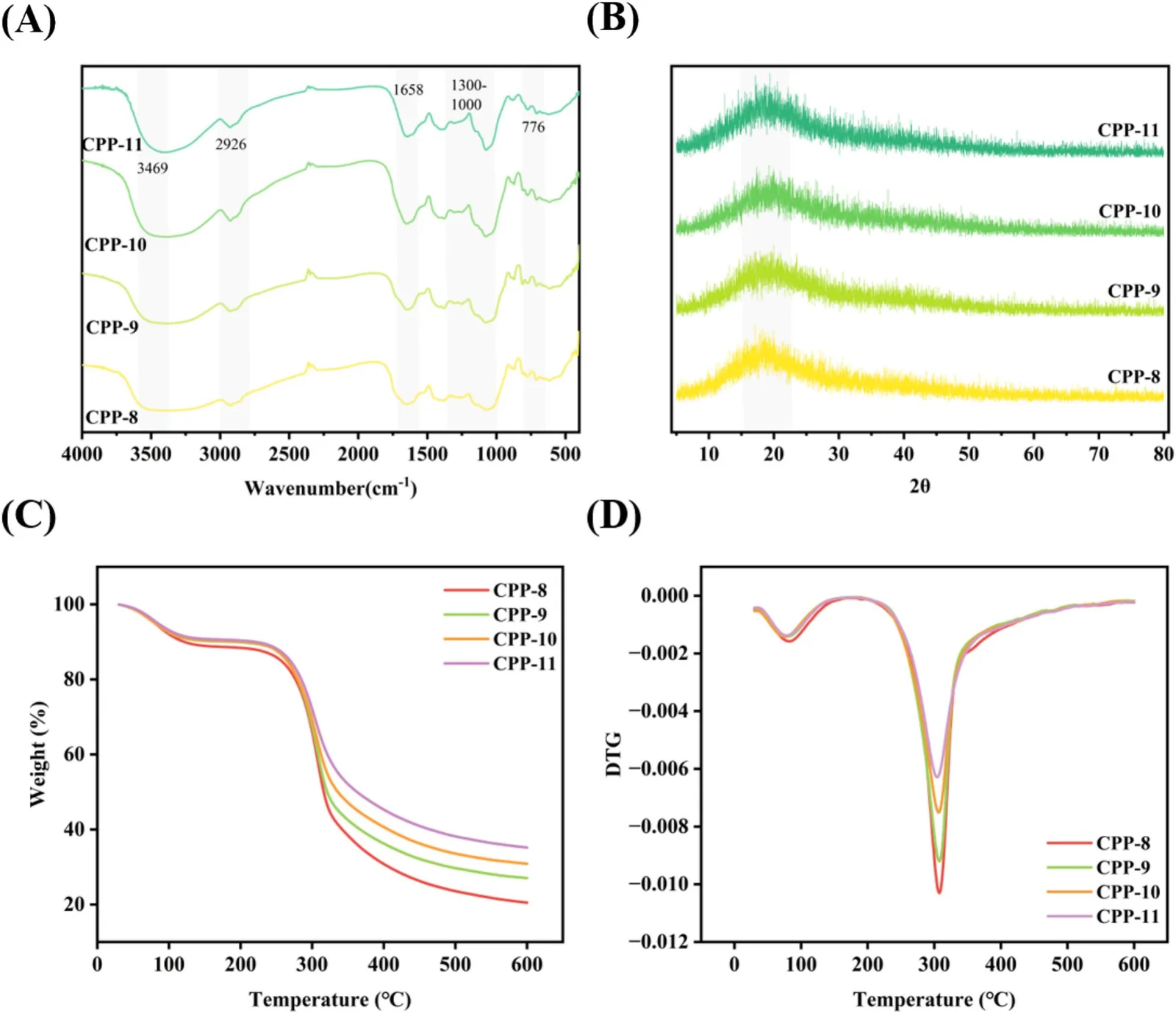
The FTIR spectra (A), XRD analysis (B), TG (C) and DTG (D) of CPP NPs prepared at different pH conditions.

### 3.2 Interfacial properties of CPP dispersion in aqueous phase as affected by extracting pH conditions

Compared with other properties, the physical properties such as zeta-potential (Fig. 2A) and hydrodynamic size (Fig. 2B) of CPP in the aqueous phase are more important when considering its use as a carrier for flavors and aromas (Goubet et al., 1998). As shown in Fig. 2B, CPP mainly existed in water as nanoparticles ranging from 200 to 250 nm. Changes in pH did not have a particularly significant effect on particle size. All CPP particles exhibited negative zeta potentials, with CPP-11 showing a significantly more negative charge than the others. It is generally believed that a greater absolute value of the zeta potential indicates better particle stability, as stronger electrostatic repulsion helps reduce intermolecular aggregation (Xie et al., 2017). The significantly more negative charge of CPP-11 was mainly attributed to its higher protein content, as shown in Table 1.

**Fig. 2:**
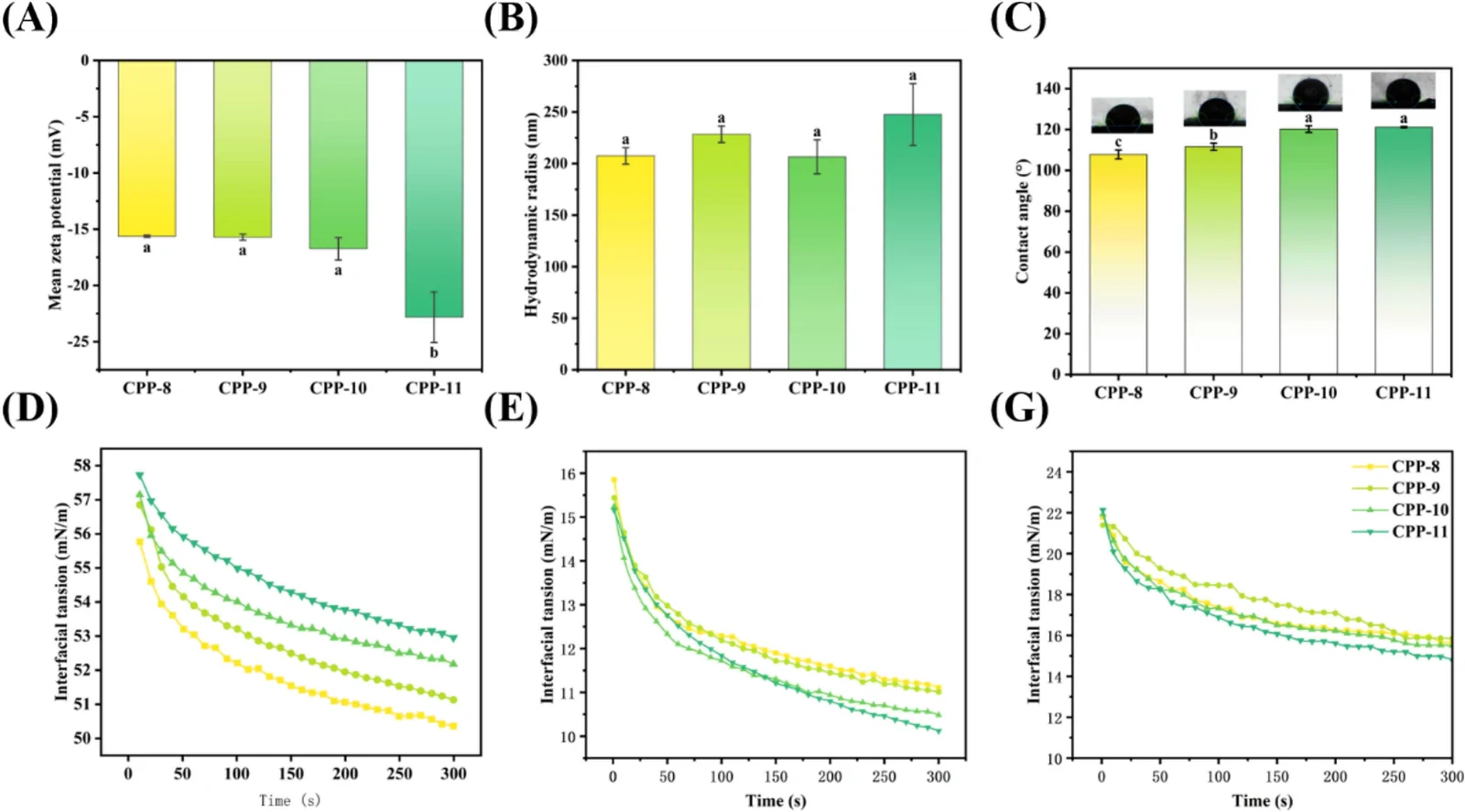
The zeta-potential (A) and particle size (B) of CPP NPs at 0.1 mg/mL in aqueous dispersion were evaluated using the DLS technique, while the three-phase contact angle (C) and dynamic interfacial tension of air/water (D), fish oil/water (E) and soybean oil/water (F) as stabilized by CPP NPs at 2 mg/mL as a function of time.

In addition to surface potential, the wettability of particles plays a crucial role in influencing the formation and properties of Pickering emulsions when these particles are used to prepare such emulsions (Lam et al., 2014). Partially wettable particles enhance the adsorption, aggregation, and stacking of colloidal particles at the interface, thereby forming a steric barrier that hinders aggregation. As shown in Fig. 2C, all CPP particles had contact angles greater than 90°, indicating that they were hydrophobic particles. Such particles are more likely to aggregate at the oil–water interface and exhibit higher desorption energy. The contact angle of CPP samples increased with increasing extraction pH, indicating enhanced hydrophobicity. This trend may reflect the higher protein content and greater exposure of hydrophobic protein groups onto the surface under strongly alkaline extraction conditions. Increased exposure of these groups may enhance particle hydrophobicity and facilitate adsorption of the CPP components at the oil–water interface (Feng, Guo, et al., 2023).

In order to better understand the interfacial behavior of CPP NPs, the dynamic interfacial tension at air/water, fish oil/water and soybean oil/water interfaces have been studied. As shown in Fig. 2D–F, The CPP NPs showed a similar trend in reducing interfacial tension at three different interfaces. The interfacial tension gradually decreased with time, indicating that CPP molecules gradually adsorbed onto the oil/water or air/water interface, and molecular rearrangement formed a stable interfacial layer (Lv et al., 2024). As a result, CPP may exhibit different conformations or orientations at these two interfaces. CPP NPs extracted at lower pH values had lower molecular weights and less negative surface charges. These properties may facilitate more rapid migration of CPP macromolecules toward the air–water interface (Han et al., 2019). No significant differences were observed among the CPP samples at the soybean oil–water interface. The polarity of soybean oil lies between those of air and fish oil. This observation further supports the proposed explanation for the interface-dependent behavior of CPP NPs.

To further study the interfacial tension lowering properties, the dynamic interfacial tension of the soybean oil/water interface in the presence of CPP at different concentrations was studied. The initial interfacial tension (γ_₀_) and the estimated equilibrium interfacial tension (γ_∞_), calculated from the intercept of the linear plot of the instantaneous γ versus 1/$\sqrt{t}$, are summarized in Table 2 (Zhang & Romero, 2020).

**Table 2:** The initial interfacial tension (γ_0_) of CPP-stabilized soybean oil/water mixture and the estimated interfacial tension (γ_∞_) at equilibrium state.

	γ(mN/m)	CPP-8	CPP-9	CPP-10	CPP-11
2 mg/mL	γ_0_	22.18 ± 0.17^Ab^	23.30 ± 0.13^Aa^	23.13 ± 0.29^Aa^	23.36 ± 0.31^Aa^
	γ_∞_	14.78 ± 0.06^Ad^	17.43 ± 0.23^Ab^	16.37 ± 0.19^Ac^	18.03 ± 0.30^Aa^
6 mg/mL	γ_0_	20.23 ± 0.24^Bb^	21.87 ± 0.07^Bab^	20.99 ± 0.11^Bb^	22.76 ± 1.32^ABa^
	γ_∞_	14.01 ± 0.11^Bc^	15.53 ± 0.04^Ba^	14.82 ± 0.01^Bb^	15.78 ± 0.23^Ba^
10 mg/mL	γ_0_	19.35 ± 0.12^Cb^	19.25 ± 0.64^Cb^	19.46 ± 0.05^Cb^	20.79 ± 0.14^Ba^
	γ_∞_	13.05 ± 0.21^Cb^	12.73 ± 0.88^Cb^	13.37 ± 0.10^Cab^	14.56 ± 0.01^Ca^

Note: Values are presented as mean ± SD (*n* = 3). Uppercase letters indicate significant differences in γ_₀_ or γ_∞_ within the same column among CPP concentrations, whereas lowercase letters indicate significant differences among CPP samples at the same concentration across different extraction pH values (same row), as determined by one-way ANOVA followed by Tukey's multiple-comparison test (*p* < 0.05).

The initial interfacial tension decreased with increasing CPP concentration, indicating that the CPP concentration had not reached saturation within the tested range (*p* < 0.05). Therefore, as the CPP concentration increased, adsorption at the interface accelerated, leading to a decrease in interfacial tension. Among the CPP samples extracted at different pH values, significant differences were also observed in the γ_₀_ values, and the estimated γ_∞_ values. At 10 mg/mL, the γ_₀_ value of CPP-11 (20.79 ± 0.14 mN/m) was significantly higher than those of CPP-8 (19.35 ± 0.12 mN/m), CPP-9 (19.25 ± 0.64 mN/m), and CPP-10 (19.46 ± 0.05 mN/m). Similarly, the γ_∞_ value of CPP-11 (14.56 ± 0.01 mN/m) was significantly higher than those of CPP-8 (13.05 ± 0.21 mN/m) and CPP-9 (12.73 ± 0.88 mN/m), whereas CPP-10 (13.37 ± 0.10 mN/m) showed an intermediate value. The results may indicate that the CPP-11 sample was less effective to be adsorbed at the interface, but the newly formed interface by CPP-11 has higher resistance to interfacial deformation (Feng, Zhang, et al., 2023). These results indicate that the interfacial tension-reducing ability of CPP was affected by both extraction pH and CPP concentration, although the effect of extraction pH was not monotonic.

### 3.3 Emulsifying properties of CPP NPs as affected by extracting pH conditions

As shown in Fig. 3A and B, for fish oil emulsions, the droplet size of emulsions stabilized by CPP extracted at higher pH values was larger than that of emulsions stabilized by CPP obtained under lower-pH conditions, whereas the freshly prepared soybean oil emulsions showed no significant differences among the samples. When comparing the zeta potential values (Fig. 3C and D), the absolute zeta potential of CPP-stabilized fish oil emulsions increased during storage, especially for the CPP-11-stabilized fish oil emulsion, which increased from −36.17 ± 0.88 mV on day 0 to −42.56 ± 0.80 mV on day 3. This result indicates enhanced interparticle repulsion, suggesting that interfacial rearrangement may still occur during storage. However, all CPP-stabilized emulsions exhibited good stability, with no significant differences in droplet size within 7 days (except for CPP-10), indicating that the CPP composite dispersions exhibited good emulsifying and stabilizing properties. For soybean oil emulsions, no significant differences were observed among the zeta potential values of the samples. But emulsions stabilized by CPP-10 and CPP-11 exhibited more stable droplet sizes during storage.

**Fig. 3:**
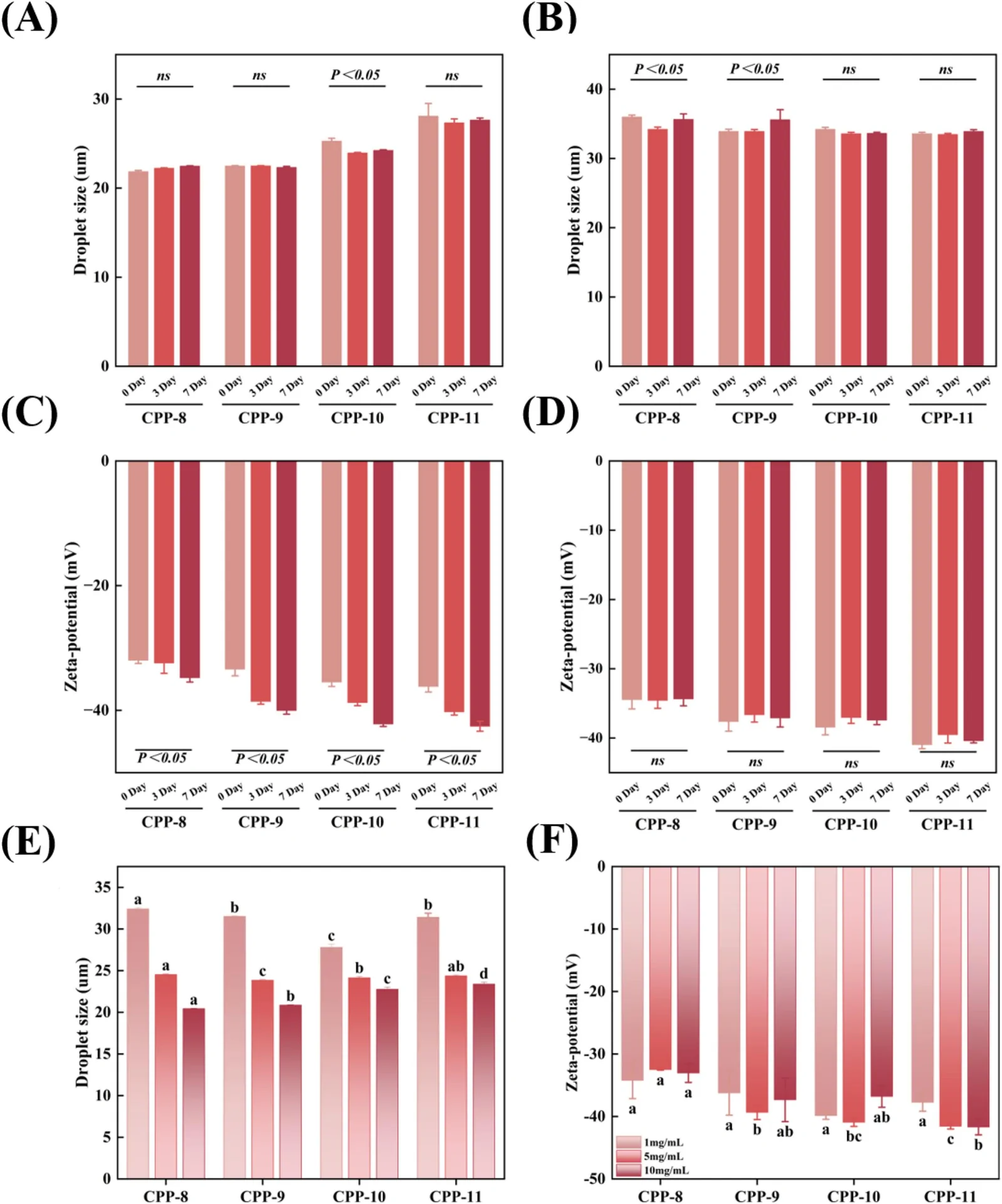
The droplet size (A–B) and zeta potential (C–D) of fish oil (A,C) and soybean oil (B,D) emulsions stabilized by CPP NPs within seven days; E and F show the impact of CPP concentration on the droplet size and zeta potential of soybean oil emulsion, respectively. (a–c) indicate significant differences among samples (*p* < 0.05). Note: Values are presented as mean ± SD (*n* = 3). In panels A–D, *p* < 0.05 indicates a significant overall difference among storage times, whereas “ns” indicates no significant difference. In panels E and F, different lowercase letters indicate significant differences among CPP concentrations within the same CPP sample (*p* < 0.05).

To further investigate the effect of different CPP concentrations on emulsion properties, soybean oil emulsions prepared with varying CPP concentrations were evaluated. The results showed that increasing the CPP concentration gradually decreased the emulsion droplet size (Fig. 3E). This reduction could be attributed to the fact that higher CPP concentration can better reduce the interfacial tension between water and oil and form a relatively thicker layer on the droplet surface, thereby inhibiting droplet aggregation and maintaining uniformity. However, CPP concentration did not significantly affect the zeta potential of the emulsions. This result suggests that the droplet surface charge was already saturated or was not governed primarily by the adsorbed CPP NPs (Wang & Zhang, 2019). In addition, no obvious trend in droplet size or zeta potential was observed among samples containing CPP NPs prepared under different extraction pH conditions, which may be consistent with the observation above that the interfacial tension-reducing ability of CPP was not significantly affected by the extraction pH conditions. Although DLS results confirmed the presence of a major nanoscale particle population of approximately 200–250 nm, the CPP fraction was not further separated into particulate and soluble components. Therefore, the observed emulsion stability should be attributed to the CPP composite dispersion as a whole rather than exclusively to the nanoparticles, because soluble polysaccharide–protein complexes and other co-extracted components may also contribute through interfacial adsorption or modification of the continuous phase properties.

### 3.4 Multi-flavor formulations stabilized by CPP NPs

Although CPP-11 did not exhibit the lowest interfacial tension in every comparison, it showed the highest extraction yield (7.00 ± 0.64%; *p* < 0.05), a significantly more negative particle zeta potential than the other CPP samples, and good emulsion stability, as indicated by the absence of significant changes in droplet size during 7 days of storage in both fish-oil and soybean-oil emulsions (*p* > 0.05). Considering these parameters collectively, CPP-11 was selected for subsequent flavor-loading experiments. The six flavor products were chosen because of their common use in cigarette-related products and their relevance to thermally processed applications. Their storage stability was subsequently evaluated by measuring particle size and zeta potential on days 0, 3, and 7, as shown in Fig. 4.

**Fig. 4:**
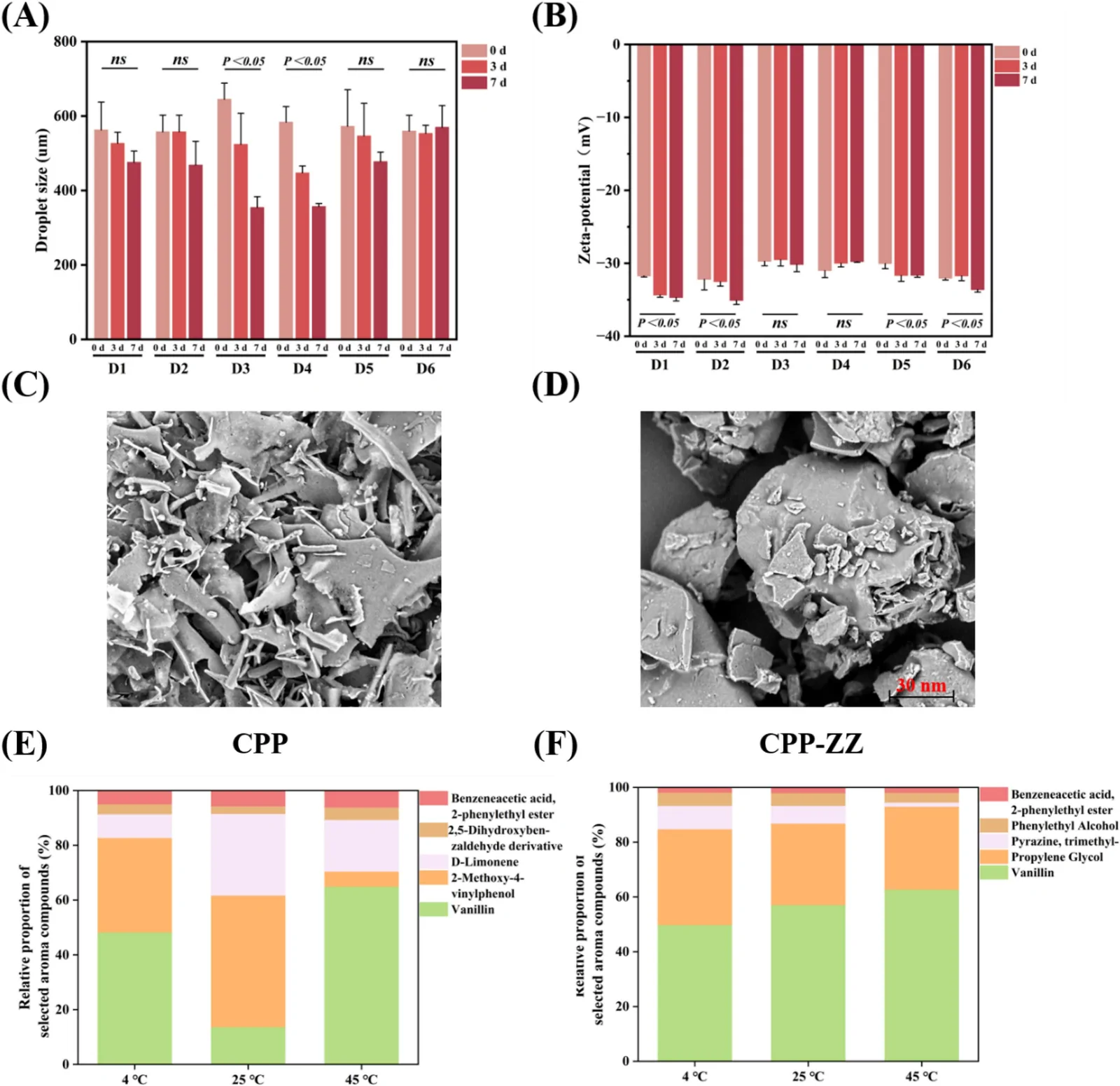
The droplet size (A) and zeta potential (B) of six commercial flavor emulsions stabilized by CPP NPs within seven days; (C) and (D) show the SEM images of lyophilized powders of CPP NPs and CPP NP-stabilized cocoa flavor extract (D5) emulsion, respectively; Main VOCs released from lyophilized CPP NPs (E) and CPP NP-stabilized cocoa flavor extract emulsion (D5) (F), respectively. Note: Panels C and D were acquired at the same magnification and are presented at the same scale. The scale bar shown in panel D also applies to panel C.

The particle sizes of the emulsion samples ranged from 560 to 645 nm (Fig. 4A). A more stable particle size during storage indicates better colloidal stability and, consequently, more stable performance. With increasing storage time, a slight decrease in particle size was observed, particularly for emulsion samples with D3 and D4, which exhibited a significant reduction after 7 days of storage (*p* < 0.05). This phenomenon may be attributed to changes in interactions among certain components within the system, potentially resulting from the rearrangement of CPP NPs at the oil–water interface (Bon, 2014). In contrast, the particle sizes of samples D1, D2, D5, and D6 showed no statistically significant changes during storage (*p* > 0.05), indicating that their physical structures, or the CPP-stabilized interfacial structures, remained relatively stable throughout the storage period.

The zeta potentials of all formulations were also monitored during storage. D1, D2, D5, and D6 exhibited significant increases in absolute zeta potential over the storage period (*p* < 0.05). The resulting enhancement in electrostatic repulsion may inhibit particle aggregation and improve emulsion stability. In contrast, the absolute zeta potentials of D3 and D4 did not change significantly during storage. This finding indicates that their electrostatic repulsion remained relatively unchanged during storage. Quantitatively, the zeta potential values of D1, D2, D5, and D6 changed from −31.74 ± 0.15, −32.21 ± 1.43, −30.05 ± 0.68, and − 32.06 ± 0.24 mV on day 0 to −34.71 ± 0.46, −35.10 ± 0.55, −31.67 ± 0.25, and − 33.62 ± 0.34 mV on day 7, respectively (*p* < 0.05). By comparison, the zeta potentials of D3 and D4 changed from −29.73 ± 0.62 and − 31.01 ± 0.96 mV to −30.17 ± 0.98 and − 29.77 ± 0.09 mV, respectively, without statistically significant differences (*p* > 0.05). Although D5 did not exhibit the most negative zeta potential among the formulations, its absolute zeta potential remained relatively stable during storage, changing from 30.05 ± 0.68 mV on day 0 to 31.67 ± 0.25 mV on day 7 (*p* < 0.05). In addition, the particle size did not change significantly, from 572.41 ± 98.45 to 477.62 ± 25.64 nm (*p* > 0.05), indicating great physical stability over the 7-day storage period. Therefore, D5 was selected for subsequent experiments primarily based on its favorable emulsion stability and the representative characteristics of its complex flavor composition (discussed in the following section).

Fig. 4C shows the SEM images of lyophilized powders of CPP NPs and CPP NP-stabilized cocoa flavor extract (D5) emulsion. The lyophilized CPP sample exhibited a sheet-like or lamellar morphology with relatively rough and heterogeneous surfaces. A small number of fibrous or curled chain-like structures were observed, and distinct pores were present between the sheet-like structures, resulting in an overall loose architecture. In contrast, D5 powder sample exhibited a more compact aggregated and quasi-spherical morphology with a smoother and more uniform surface appearance. Fragmented sheet-like structures were markedly reduced, and the particle edges became smoother and more rounded, indicating enhanced intermolecular interactions within the system.

The antioxidant activity of CPP stabilized D5 (CPP-D5) was compared with that of CPP alone and free cocoa flavor (Free D5) before and after temperature treatment (Table S2). After heating, CPP stabilized D5 exhibited a DPPH radical-scavenging activity of 30.21 ± 0.55%, compared with 9.20 ± 0.57% for Free D5 and 13.65 ± 0.19% for CPP alone (*n* = 3). Notably, the antioxidant activity of CPP stabilized D5 was substantially higher than that of either Free D5 or CPP alone and was not simply the additive effect of the two components. These results suggest that CPP stabilized D5 retained a higher overall antioxidant activity after heating. Although the DPPH assay does not directly quantify aroma retention or release behavior, these results provide supportive evidence that the carrier system may help preserve the functional properties associated with D5 during thermal treatment.

### 3.5 Aroma characteristics of the CPP-D5 formulation as affected by storage

According to the HS-SPME-GC–MS results in Table S1, multiple volatile compounds were detected in both CPP NPs and CPP-D5 samples, including phenols, aldehydes, alcohols, esters, and pyrazines. Similar classes of aroma-active volatile compounds have recently been identified in coffee products using HS-SPME-GC–MS (Zhai et al., 2024), supporting the relevance of the volatile profiles observed in the present study. The major volatile compounds detected in CPP NPs included 2-methoxy-4-vinylphenol, phenylethyl phenylacetate, cinnamaldehyde, 2,4-di-tert-butylphenol, and vanillin. The high abundance of vanillin, which is also a common volatile compound found in roasted coffee, may result from degradation of lignin in SCG. Among these compounds, 2-methoxy-4-vinylphenol is generally associated with smoky, spicy, and phenolic aroma notes, while cinnamaldehyde and phenylethyl phenylacetate may contribute warm cinnamon-like, floral, and honey-like notes (Zakidou et al., 2021). It should also be noted that the volatile compounds identified above, as key coffee aroma compounds, mainly contribute to the fruity and floral characteristics of coffee. However, coffee aroma is highly complex and also includes roasted and nutty aroma compounds, such as 2-furfurylthiol, which is considered one of the key compounds contributing to the characteristic aroma of freshly brewed coffee. These compounds were not detected in the aroma profile of CPP NPs, which may be attributed to the alkaline extraction process of CPP, as it was primarily focused on the protein–polysaccharide matrix. Therefore, the potential contribution of compounds such as 2,4-di-tert-butylphenol and dibutyl terephthalate might also originate from background or protein–polysaccharide matrix.

Compared with CPP NPs alone, D5 exhibited a more diverse volatile profile, indicating that the incorporation of cocoa flavor extract enriched the aroma composition of the system. This broader volatile profile likely resulted from the combination of intrinsic SCG-derived volatiles and compounds introduced by the cocoa flavor extract, rather than from the generation of new compounds during storage.

The effects of storage temperature on the relative composition of selected major VOCs are shown in Fig. 4E and F. For CPP NPs, 2-methoxy-4-vinylphenol was the dominant compound at 4 °C, whereas its relative proportion decreased markedly at 25 °C and 45 °C. In contrast, the relative proportion of vanillin increased at 45 °C, suggesting that the aroma profile of CPP NPs shifted from phenolic/smoky notes toward a relatively sweeter vanilla-like profile under elevated-temperature storage. For D5 samples, vanillin remained the dominant volatile compound across all storage temperatures, followed by propylene glycol, phenylethyl alcohol, and trimethylpyrazine. Trimethylpyrazine and phenylethyl alcohol are associated with roasted, nutty, floral, and honey-like aroma characteristics, whereas propylene glycol may originate from the commercial cocoa flavor extract as a carrier component (Spizzirri et al., 2019). Although the relative proportions of the selected VOCs changed with increasing temperature, vanillin remained at a relatively high proportion in D5, indicating that D5 maintained a vanilla-like aroma profile after storage. Because the results were expressed as normalized relative peak areas, an increase in the relative proportion of one compound may partly reflect decreases in other compounds. Therefore, these results indicate temperature-dependent changes in the volatile composition rather than absolute flavor retention. This interpretation is consistent with Gantner et al. (2024), who reported that lower storage temperatures slowed changes in coffee volatile profiles and better preserved desirable aroma characteristics.

### 3.6 Pyrolysis analysis of volatile components in the CPP-D5 formulation at various temperatures

Considering the potential application of the CPP NPs powder formulation in cigarette-related thermal applications, Py-GC–MS was performed to characterize the thermal behavior and relative abundance of volatile compounds from the formulation as a whole, rather than attributing all detected volatiles solely to the release of encapsulated flavors. Therefore, the pyrolysis-GC/MS analysis was conducted to investigate the thermal degradation behavior of lyophilized CPP NPs and CPP NP-stabilized cocoa flavor extract (D5) at 200, 275, and 350 °C (Table 3). At 200 °C, D5 was dominated by cocoa extract-derived flavor compounds (Chung & Iiyama, 2003), particularly vanillin (68.67%) and propylene glycol (23.99%). In contrast, CPP NPs without flavor extract generated substantial amounts of carbon dioxide (47.05%), methyl stearate (28.82%), and methyl hexadecanoate (24.13%) at 200 °C, indicating the initial thermal decomposition of polysaccharide and lipid components within the nanoparticle matrix (Liu et al., 2016). The much higher abundance of vanillin and propylene glycol in D5 suggests that the encapsulated cocoa flavor compounds remained largely intact at this temperature and were gradually released rather than extensively degraded. As the temperature increased to 275 °C, significant degradation of carbohydrate components was observed in CPP NPs, as evidenced by the formation of levoglucosan (52.57%), lactose-derived products (14.36%), and 5-hydroxymethylfurfural. These compounds are characteristic pyrolysis products generated through dehydration and depolymerization reactions of polysaccharides (Peng et al., 2025; Shafizadeh, 1982). In contrast, D5 still retained detectable levels of vanillin (47.77%) and 2-phenylethyl phenylacetate (1.62%), two key aroma compounds associated with sweet, floral, and cocoa-like sensory notes. The persistence of these flavor compounds at elevated temperatures suggests that interactions between the flavor molecules and the polysaccharide–protein matrix may have delayed their thermal degradation, potentially contributing to greater flavor complexity during heating. Further heating to 350 °C resulted in a profile characterized by extensive fragmentation and secondary reactions. The appearance of small carbonyl compounds (e.g., hydroxyacetone, methylcyclopentanone, and heptanal) is consistent with advanced thermal fragmentation reactions of carbohydrate-containing matrices (Peng et al., 2025). Overall, the results suggest that cocoa-related compounds remain detectable in D5 during pyrolysis, although the normalized relative peak areas do not directly quantify their absolute thermal retention. The relative abundance of vanillin in D5 decreased from 68.67% at 200 °C to 47.77% at 275 °C and 24.29% at 350 °C, accompanied by an increase in smaller pyrolysis products at the elevated temperatures. In CPP NPs, levoglucosan became the predominant product at 275 and 350 °C, indicating progressive carbohydrate dehydration and depolymerization during heating (Peng et al., 2025). These results demonstrate temperature-dependent changes in the relative composition of the pyrolysis products.

**Table 3:** The volatile pyrolysis products of lyophilized CPP NPs and CPP NP-stabilized cocoa flavor extract (D5) at 200, 275 and 350 °C.

Compound	Formula	Chemical class	D5 200 °C (%)	CPP 200 °C (%)	D5 275 °C (%)	**CPP 275 °C (%)**	**D5 350 °C (%)**	**CPP 350 °C (%)**
(*R*)-3-Methylcyclopentanone	C_6_H_10_O	Carbonyl compound	ND	ND	ND	ND	ND	0.31
2,4-Dimethyl-3-hexanol	C_8_H_18_O	Alcohol/polyol	ND	ND	ND	ND	ND	6.51
2-Deoxy-d-ribose	C_5_H_10_O_4_	Sugar-derived/furan	ND	ND	21.80	ND	10.54	ND
2-furfuryl alcohol	C_5_H_6_O_2_	Sugar-derived/furan	ND	ND	2.59	ND	ND	ND
2-Hydroxy-3-methyl-2-cyclopenten-1-one	C_6_H_8_O_2_	Others	ND	ND	ND	ND	0.61	ND
2-Hydroxymethyl-2-nitro-1,3-propanediol	C_4_H_9_NO_5_	Nitrogen-containing	ND	ND	ND	ND	ND	2.97
2-Phenylethyl phenylacetate	C_16_H_16_O_2_	Acid/ester/lipid-derived	ND	ND	1.62	ND	0.56	ND
2-Propanone	C_3_H_6_O	Carbonyl compound	ND	ND	ND	ND	ND	0.13
3-Nonanol	C_9_H_20_O	Alcohol/polyol	ND	ND	ND	ND	ND	4.22
3-Heptanol	C_7_H_16_O	Alcohol/polyol	ND	ND	ND	3.98	ND	ND
3-Hexanol	C_6_H_14_O	Alcohol/polyol	ND	ND	ND	ND	ND	2.08
3-Isopropyl-2,5-piperazinedione	C_7_H_12_N_2_O_2_	Nitrogen-containing	ND	ND	ND	ND	ND	0.27
3-Methylcyclopentane-1,2-diol	C_6_H_12_O_2_	Others	ND	ND	ND	9.42	0.81	ND
5-hydroxymethylfurfural	C_6_H_6_O_3_	Sugar-derived/furan	ND	ND	ND	2.06	ND	0.33
9,12-Octadecadienoic acid	C_18_H_32_O_2_	Acid/ester/lipid-derived	ND	ND	ND	ND	ND	0.42
Acetic acid	C_2_H_4_O_2_	Acid/ester/lipid-derived	ND	ND	ND	ND	1.33	ND
Carbon dioxide	CO_2_	Gas/inorganic	5.88	47.05	1.43	1.46	18.97	5.62
Cyclopropylmethanol	C_4_H_8_O	Alcohol/polyol	ND	ND	ND	ND	2.19	ND
Ethyl 3-hydroxyhexanoate	C_8_H_16_O_3_	Others	ND	ND	ND	ND	ND	1.01
Ethylene glycol	C_2_H_6_O_2_	Alcohol/polyol	ND	ND	ND	ND	0.20	ND
Glyceryl Monopalmitate	C_35_H_68_O_5_	Acid/ester/lipid-derived	ND	ND	ND	ND	0.28	ND
Guaiacol	C_7_H_8_O_2_	Phenolic/aromatic	ND	ND	0.65	ND	0.27	ND
H-Pyran-4-one	C_6_H_8_O_4_	Sugar-derived/furan	ND	ND	ND	ND	ND	0.27
Hydroxyacetone	C_3_H_6_O_2_	Carbonyl compound	ND	ND	ND	ND	4.79	0.61
Isosorbide	C_6_H_10_O_4_	Sugar-derived/furan	ND	ND	ND	ND	0.35	ND
Lactose	C_12_H_22_O_11_	Sugar-derived/furan	ND	ND	ND	14.36	ND	4.16
Levoglucosan	C_6_H_10_O_5_	Sugar-derived/furan	ND	ND	ND	52.57	7.44	36.24
Methanol	CH_3_OH	Alcohol/polyol	ND	ND	ND	1.82	ND	ND
Methyl formate	C_2_H_4_O_2_	Acid/ester/lipid-derived	ND	ND	1.38	ND	12.65	13.89
Methyl hexadecanoate	C_17_H_34_O_2_	Acid/ester/lipid-derived	0.24	24.13	ND	4.55	ND	ND
Methyl oleate	C₁₉H₃₆O₂	Acid/ester/lipid-derived	0.76	ND	ND	ND	ND	ND
Methyl pyruvate	C_4_H_6_O_3_	Acid/ester/lipid-derived	ND	ND	ND	ND	ND	0.37
Methyl stearate	C_19_H_38_O_2_	Acid/ester/lipid-derived	0.46	28.82	0.68	0.57	ND	0.07
Methylcyclopentanone	C_6_H_10_O	Carbonyl compound	ND	ND	ND	ND	0.69	ND
Methylglyoxal	C_3_H_4_O_2_	Carbonyl compound	ND	ND	ND	ND	8.55	11.17
n-Heptanal	C_7_H_14_O	Carbonyl compound	ND	ND	ND	ND	ND	0.73
o-cresol	C_7_H_8_O	Phenolic/aromatic	ND	ND	8.70	ND	ND	ND
Oleic acid	C_18_H_34_O_2_	Acid/ester/lipid-derived	ND	ND	ND	ND	0.13	ND
Palmitic acid	C_16_H_32_O_2_	Acid/ester/lipid-derived	ND	ND	0.42	6.41	1.84	1.63
Pentanal	C_5_H_10_O	Carbonyl compound	ND	ND	ND	2.80	ND	ND
Pineapple ketone	C_6_H_8_O_3_	Carbonyl compound	ND	ND	ND	ND	ND	0.39
Propylene glycol	C_3_H_8_O_2_	Alcohol/polyol	23.99	ND	5.21	ND	0.83	ND
Stearic acid	C_18_H_36_O_2_	Acid/ester/lipid-derived	ND	ND	2.68	ND	1.54	0.89
Theobromine	C_7_H_8_N_4_O_2_	Nitrogen-containing	ND	ND	3.17	ND	ND	ND
Trimethylamine	C_3_H_9_N	Nitrogen-containing	ND	ND	1.89	ND	ND	5.53
Tris(hydroxymethyl)nitromethane	C_4_H_9_NO_5_	Nitrogen-containing	ND	ND	ND	ND	1.06	ND
Vanillin	C_8_H_8_O_3_	Phenolic/aromatic	68.67	ND	47.77	ND	24.29	ND
Z9–9, Z9–12-Octadecadienoic acid	C₁₈H₃₂O₂	Acid/ester/lipid-derived	ND	ND	ND	ND	ND	0.18

Note: Compounds are arranged alphabetically according to their standardized names. ND indicates not detected. Relative abundance (%) was calculated by dividing the peak area of each compound by the total peak area of all detected compounds in the same chromatogram and multiplying by 100. Synonymous compound names were merged; when synonymous entries occurred in the same chromatogram, their relative abundances were summed. Standardized merged names: Propylene glycol (including 1,2-propanediol); Methyl hexadecanoate (including methyl palmitate); Palmitic acid (including n-hexadecanoic acid and hexadecanoic acid); Stearic acid (including octadecanoic acid); Guaiacol (including o-methoxyphenol); Levoglucosan (including 1,6-dehydrated-β-D-pyranose glucose); 2-Phenylethyl phenylacetate; Methylglyoxal (including acetone aldehyde); Hydroxyacetone (including 1-hydroxy-2-propanone); 3-Methylcyclopentane-1,2-diol; and 2-Deoxy-d-ribose (including 2-deoxy-D-erythro-pentose).

## 4 Conclusion

Endogenous polysaccharide–protein complexes recovered from spent coffee grounds were successfully transformed into functional nanoparticles capable of encapsulating and delivering flavor extracts. The pH applied during the alkaline extraction process significantly influenced the composition and physicochemical properties of the resulting CPP NPs. Among the formulations investigated, CPP NPs prepared at pH 11 were selected based on their favorable physicochemical properties and storage stability and were subsequently used to produce the freeze-dried flavor powder. Cocoa-related volatile compounds remained detectable in the flavor-loaded powder after storage, while its normalized volatile profile changed with storage and pyrolysis temperatures. These findings highlight the potential of naturally occurring polysaccharide–protein composites as multifunctional delivery systems that integrate structural support, flavor protection, and thermally triggered aroma release. Beyond cocoa flavor delivery, CPP NPs could potentially serve as carriers for lipophilic flavor and aroma compounds in a range of applications, particularly tobacco and cigarette products, where the controlled release of aroma compounds during thermal processing or smoking could offer opportunities for flavor modulation. Their emulsifying ability and compatibility with freeze-dried powder preparation may facilitate their incorporation into dry premixes and food emulsions. Their potential applicability may also extend to bakery and confectionery products, instant beverage powders, and other emulsion-based or thermally processed food products. Nevertheless, product-specific sensory properties, processing stability, safety, and regulatory compliance should be further evaluated before their practical application in the food industry. More importantly, this work demonstrates a high-value utilization strategy for SCG through the development of waste-derived flavor delivery materials.

## CRediT authorship contribution statement

**Haitao Tao:** Writing – original draft, Data curation. **Weilong Guan:** Writing – review & editing, Data curation. **Haibo Zhu:** Writing – review & editing, Data curation. **Qiuyu Han:** Data curation. **Mingyang Dai:** Writing – review & editing, Data curation. **Xin Gao:** Writing – review & editing, Data curation. **Yawen Lin:** Writing – review & editing, Project administration, Funding acquisition. **Yue Zhang:** Writing – review & editing, Project administration, Conceptualization.

## Declaration of competing interest

The authors declare that they have no known competing financial interests or personal relationships that could have appeared to influence the work reported in this paper.

## Data Availability

Data will be made available on request.
