# Supplementary material for: Endogenous polysaccharide–protein composite nanoparticles prepared from spent coffee grounds as multi-flavor carriers
Source: Food Chem X. 2026 Oct 15;40:104636. doi: 10.1016/j.fochx.2026.104636 (PMC13691143; doi:10.1016/j.fochx.2026.104636)
Supplement: Supplementary file 1 [file mmc1.docx]

**Supplementary data**

**Table S1 The impact of storage on VOCs released from lyophilized CPP NPs and CPP NP stabilized cocoa flavor extract (CPP-D5) determined by HS-SPME-GC–MS.**

| Compound | Formula | 4 °C CPP-D5 (%) | 25 °C CPP-D5 (%) | 45 °C CPP-D5 (%) | 4 °C CPP (%) | 25 °C CPP (%) | 45 °C CPP (%) |
| --- | --- | --- | --- | --- | --- | --- | --- |
| Vanillin | C_8_H_8_O_3_ | 51.29 | 52.43 | 60.83 | 37.32 | 9.56 | 53.61 |
| Propylene Glycol | C_3_H_8_O_2_ | 31.97 | 30.76 | 29.40 | — | — | — |
| Pyrazine, trimethyl- | C_7_H_10_N_2_ | 7.78 | 6.70 | 1.49 | — | — | — |
| Phenylethyl Alcohol | C_8_H_10_O | 4.41 | 4.80 | 3.47 | — | — | — |
| Phenylethyl Phenylacetate | C_16_H_16_O_2_ | 1.65 | 2.07 | 1.80 | 3.83 | 3.97 | 5.01 |
| Cinnamaldehyde | C_9_H_8_O | 0.74 | 0.72 | 0.37 | 9.60 | 8.59 | 4.86 |
| Vanillin propylene glycol acetal | C_11_H_14_O_4_ | 0.72 | 0.63 | 1.00 | — | — | — |
| Butanoic acid, 3-methyl- | C_5_H_10_O_2_ | 0.44 | 0.52 | 0.32 | — | — | — |
| 2-Propanol, 1,1'-oxybis- | C_6_H_14_O_3_ | 0.38 | 0.45 | 0.51 | — | — | — |
| 1,2-Propanediol, 1-acetate | C_5_H_10_O_3_ | 0.21 | 0.32 | 0.33 | — | — | — |
| D-Limonene | C_10_H_16_ | 0.06 | 0.23 | 0.21 | 6.71 | 20.83 | 15.59 |
| 2,4-Di-tert-butylphenol | C_14_H_22_O | 0.06 | 0.05 | 0.04 | 1.94 | 1.95 | 1.33 |
| Naphthalene | C_11_H_10_ | 0.08 | 0.07 | 0.07 | 1.72 | 4.27 | 1.43 |
| Benzene, 1,2,3,5-tetramethyl- | C_10_H_14_ | 0.10 | 0.09 | 0.05 | 3.33 | 6.51 | 4.68 |
| 2-Methoxy-4-vinylphenol | C_10_H_14_O | 0.08 | 0.08 | 0.06 | 26.66 | 33.57 | 4.55 |
| Dibutyl Terephthalate | C_16_H_22_O_4_ | 0.05 | 0.07 | 0.05 | 8.91 | 10.76 | 8.96 |

**Table S2. DPPH radical-scavenging activity of CPP, free cocoa flavor, and CPP-D5 before and after treatment for 2 h.**

| **Sample** | **Initial (0 min)** | **25 °C, 2 h** | **80 °C, 2 h** |
| --- | --- | --- | --- |
| CPP | 26.38 ± 0.52 | 18.72 ± 0.23 | 13.65 ± 0.19 |
| Free D5 | 19.02 ± 0.42 | 11.66 ± 0.45 | 9.20 ± 0.57 |
| CPP-D5 | 39.11 ± 0.18 | 40.34 ± 0.38 | 30.21 ± 0.55 |

Note: Values represent DPPH radical-scavenging activity (%) and are expressed as mean ± SD (n = 3). All samples were tested at the same total dry-sample concentration of 0.50 mg/mL after dilution. CPP, coffee-derived polysaccharide–protein complexes; Free D5 (CF), free cocoa flavor; CPP-D5, the CPP-containing cocoa flavor formulation.
